# Intractable Tangles in the Bird Family Tree

**DOI:** 10.1371/journal.pbio.1002225

**Published:** 2015-08-18

**Authors:** Roland G. Roberts

**Affiliations:** Public Library of Science, Cambridge, United Kingdom

## Abstract

Rapid sequential speciation events can outpace the fixation of genetic variants, resulting in a family tree that lacks clear branching patterns. A new study of bird genomes reveals such an explosive super-radiation that may coincide with the mass extinction at the end of the Cretaceous period.

Back in the Cretaceous period, when nonavian dinosaurs and archaic birds ruled the earth, their modern avian cousins were quietly starting to diversify. One major branch split off, giving rise to the ratites (ostriches, emus, and their kin) and tinamous, and then another—the fowls (waterfowls, gamebirds, etc.). However, what happened next is less clear. Almost all of the remaining 36 living bird lineages (together comprising more than 10,000 species in the group known as Neoaves) seem to have emerged in one go, and attempts to resolve the relationships between these branches in the deepest thicket have been challenging and seem to deliver contradictory answers. Is this because our methods just aren’t good enough, or is there something intrinsically special about this explosion of diversity?

To answer this, we need first to consider what happens to a genome when one species splits into two. The answer is often simplified—a species has a consensus genome sequence, as do its immediate descendants (the founders of the two new species), but the subsequent lack of interbreeding means that the genomes of each species are free to accumulate random mutations; the two species’ genomes then gradually diverge so that after a period of time the two sequences are readily distinguishable.

This simplification seems to work a lot of the time, because scientists can usually blindly feed gene (or genome) sequences from a set of related species into a computer and reliably recover a unique pattern of ancient divergences—a phylogenetic tree. Thus, comparing any old genes from humans, cats, and dogs will most often give you a tree in which the human splits from a common cat–dog ancestor earlier than the cat splits from the dog.

Occasionally, however, this neat discipline of phylogenetics is frustrated, and a consensus history of species can’t be reconstructed. Why might this be? Is it just because we’re looking a long way back in time or because we’re trying to resolve speciation events that happened close together? If so, surely all we need is more data?

The main complication is that the population that makes up a given species isn’t made up of clones—there’s substantial genetic variation (polymorphism) within it, and if the population is large, then the chances are that much of that variation will be inherited by both species that exist after the split. Normally the passage of time ensures that some variants (“alleles”) become universal in one population, while others will take over in the other (a process called fixation). But what if a second split occurs soon after the first, so that the two species become three or four before fixation has occurred? The original variation will become partitioned between the emerging species and only then undergo fixation, making it well-nigh impossible to disentangle the order of events. This process, called incomplete lineage sorting (ILS), can result in different parts of the genome, appearing to give incompatible versions of the phylogenetic tree. For example, ILS is known to affect one-third of genes in the human/chimpanzee/gorilla split, clustering human or chimp with gorilla rather than human with chimp [1].

A secondary complication is that with the type of genomic variations that are usually examined—single-nucleotide changes—the variations can revert relatively easily, resulting in misleading similarities or “homoplasy”. Homoplasy can muddy the phylogenetic waters as much as ILS, so in an ideal world one would want to assess a class of variation that is less prone to reversion. One possibility is that rather than examining little typos, one could follow rare genomic changes such as the dynamic insertion of chunks of selfish DNA called retrotransposons that copy and paste themselves around the genome; the premise is that while it’s easy to mistype “bat” for “cat” (or vice versa, for reversions), mistyping “catapult” for “cat” (or vice versa) is much less likely, minimizing the chances of homoplasy.

A new study in *PLOS Biology* by Alexander Suh, Linnea Smeds, and Hans Ellegren [2] applies just such a strategy to try to resolve that knottiest of phylogenetic problems—the Neoaves radiation mentioned above. The authors scanned 48 diverse bird genomes for retrotransposons, winnowing 130,000 elements from the bird-specific *hitchcock* family (named after the director of a classic Neoaves cinematic appearance) down to 2,118 widely distributed and informative representatives. Together with the sequence of their immediate genomic neighborhood, these 2,118 elements formed an exhaustive catalogue of the retrotransposon insertion events that had happened during the neoavian radiation process.

Boiling these elements down to a matrix of presence-versus-absence (i.e., an identical insertion versus an empty ancestral site) across the 48 genomes, the authors were able to construct the most parsimonious phylogenetic tree for Neoaves, which tallied well with trees derived by more traditional methods (using nuclear gene sequences [3], for example). But while 1,373 of the retrotransposon insertion events fit this tree perfectly, 745 (35%) did not; notably, most of these conflicting events were restricted to relatively few branches of the tree.

The authors argue convincingly that because these events are highly unlikely to represent homoplasies, their apparent incompatibility with the consensus family tree must have arisen through ILS. That is, in each case a population in which some individuals had a copy of the element at a given location of the genome and others did not (i.e., there was an existing presence/absence polymorphism) had split into two species, each itself a mixture of haves and have-nots. These daughter species then split in turn, before either the haves or have-nots have had the chance to predominate. Subsequent random fixation of these would then give rise to patterns that conflict with the consensus tree.

The prevalence of ILS in the bird phylogeny can be captured more accurately by representing it as a network rather than as a classic tree with simple sequential bifurcations (Fig 1). Here we can see local network-like structure in the later radiations of the “core land birds” and “core water birds” (green and blue, respectively), where 18% and 27% of *hitchcock* insertions were subject to ILS across a couple of speciation events. However, the real shocker is the deepest Neoaves radiation (red, expanded at right), which is, frankly, a bit of a mess.

**Fig 1:**
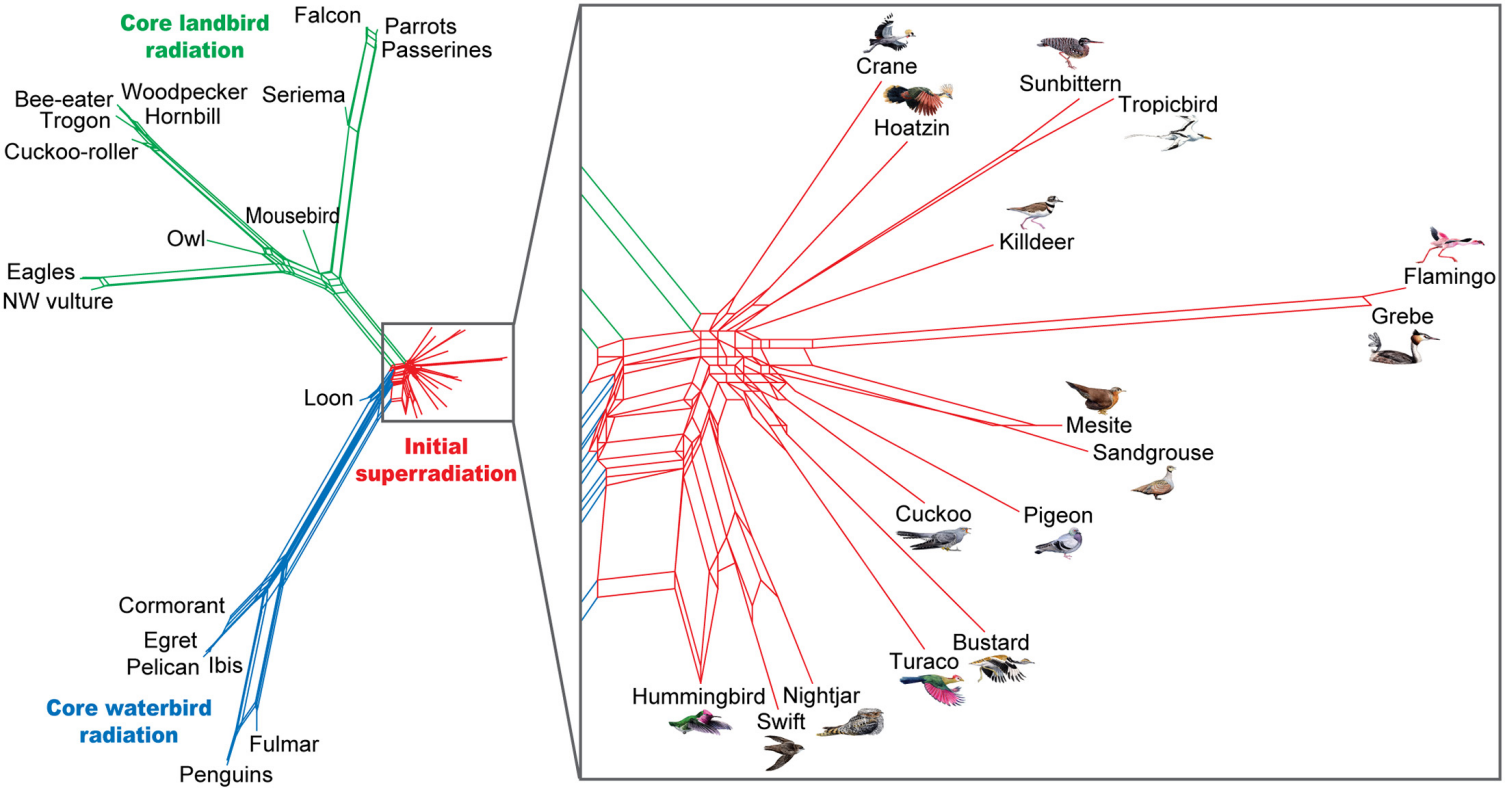
A phylogenetic network captures the complexity of adaptive radiations in the Neoaves. The early super-radiation (red), which may have occurred soon after the mass extinction event at the end of the Cretaceous period, shows spectacular degrees of incomplete lineage sorting (see expanded view at the right), testifying to the sheer explosiveness of the radiation process. *Image credit: Alexander Suh and Jon Fjeldså*.

Here in the deepest branches and shortest internodes—the thickest thicket of the tree—almost all insertion events that take place (73%) are subject to ILS, and the authors infer that most ancestral retrotransposon polymorphisms persisted through five, six, or even seven sequential speciation events (one extreme instance survived 17 events).

What is the explanation for this extraordinary behavior? From the description above of how ILS arises, it is clear that two factors will strongly predispose to ILS—short periods of time between speciation events (ensuring that fixation doesn’t have time to occur) and large effective population sizes (maximizing the chances that ancestral polymorphism will survive the split). The obvious inference from the sheer extent of ILS observed here is therefore that a large population of birds diversified very rapidly indeed.

The authors suggest that, following a recent revision of the timing of the explosive Neoaves radiation [3], something else had exploded—in a much more physical sense—immediately beforehand. Contrary to many other studies, which place the radiation firmly in the Cretaceous period, the authors’ presumption is that the spectacular adaptive radiation of the Neoaves followed hot on the heels of the destruction caused by the collision of a 10-km piece of rock with the Yucatán peninsula 66 million years ago.

This cataclysm ended the Cretaceous period by wiping out 75% of life on Earth, including all land animals weighing more than 1 kg. We could then envisage a feast of opportunity for the surviving animals—including the modern birds—scrambling to occupy a vast number of suddenly vacated ecological niches. The authors claim that the revised estimate of the timescale [3] means that the entire early ILS-riddled Neoaves super-radiation (red in Fig 1) probably took place on the clean slate of the Cretaceous–Palaeogene boundary, with the smaller (and less complex) radiations occurring within the ensuing 15 million years. A large effective population size and the near-simultaneity of isolation of the new species from each other would have resulted in the widespread inheritance of the existing repertoire of polymorphism without a chance of fixation between speciation events.

While the coincidence of the diversification of the Neoaves with the end of the dinosaurs will remain controversial (and cannot be resolved by these data alone), the evidence for pervasive ILS at the onset of the neoavian radiation is very strong and leads to the expectation that many other adaptive radiations will involve similar nonbifurcating network-like relationships occasioned by the sheer pace of speciation. Although ILS has been examined in recent (and less complex) splits, this is by far the oldest and most complex case to yield to analysis, and the retrotransposon-based approach should be applicable to others.

## Abbreviations

ILS: incomplete lineage sorting
